# Tailoring STrAtegies for RelaTives for Black and South Asian dementia family carers in the United Kingdom: A mixed methods study

**DOI:** 10.1002/gps.5868

**Published:** 2023-01-15

**Authors:** Lucy Webster, Sarah Amador, Penny Rapaport, Naaheed Mukadam, Andrew Sommerlad, Tiffeny James, Sabrina Javed, Moïse Roche, Kathryn Lord, Trishna Bharadia, Malayka Rahman‐Amin, Iain Lang, Gill Livingston

**Affiliations:** ^1^ Division of Psychiatry UCL London UK; ^2^ Centre for Health Services Studies University of Kent Canterbury UK; ^3^ Camden and Islington NHS Foundation Trust London UK; ^4^ Centre for Applied Dementia Studies University of Bradford Bradford UK; ^5^ Patient and Public Representative & Patient Author Buckinghamshire UK; ^6^ Alzheimer's Society London UK; ^7^ Exeter Medical School University of Exeter Exeter UK

**Keywords:** cultural adaption, dementia, ethnicity, family carers, mental health, psychological intervention

## Abstract

**Objectives:**

We culturally adapted STrAtegies for RelaTives (START), a clinically and cost‐effective intervention for dementia family carers, for Black and South Asian families. It had previously been delivered to family carers around the time of diagnosis, when most people with dementia had very mild, mild or moderate dementia.

**Methods:**

We interviewed a maximum variation sample of family carers (phase one; *n* = 15 South Asian; *n* = 11 Black) about what aspect of START, required cultural adaptation, then analysed it thematically using the Cultural Treatment Adaptation Framework then adapted it in English and into Urdu. Facilitators then delivered START individually to carers (phase two; *n* = 13 South Asian; *n* = 8 Black). We assessed acceptability and feasibility through the number of sessions attended, score for fidelity to the intervention and interviewing family carers about their experiences. We used the Hospital Anxiety and Depression Scale. to examine whether immediate changes in family carers' mental health were in line with previous studies.

**Results:**

In phase one we made adaptations to peripheral elements of START, clarifying language, increasing illustrative vignettes numbers, emphasising privacy and the facilitator's cultural competence and making images ethnically diverse. In phase two 21 family carers consented to receive the adapted intervention; 12 completed ≥5/8 sessions; four completed fewer sessions and five never started. Baseline HADS score (*n* = 21) was 14.4 (SD = 9.8) but for those who we were able to follow up was 12.3 (SD 8.1) and immediately post‐intervention was 11.3 (*n* = 10; SD = 6.1). Family carers were positive about the adapted START and continued to use elements after the intervention.

**Conclusions:**

Culturally adapted START was acceptable and feasible in South Asian and Black UK‐based family carers and changes in mental health were in line with those in the original clinical trial. Our study shows that culturally inclusive START was also acceptable. Changes made in adaptations were relevant to all populations. We now use the adapted version for all family carers irrespective of ethnicity.

## Key points

- Making interventions culturally tailored is important for dementia family carers from ethnic minorities.
- The culturally adapted STrAtegies for RelaTives (START) intervention was acceptable and feasible in South Asian and Black dementia family carers.
- There were also similar improvements in mental health in comparison to those in the trial of the original START intervention.

## 1 INTRODUCTION

Family members providing care to a person with dementia are often psychologically distressed, with up to 40% showing clinical levels of depression and anxiety.^1^, ^2^ We developed the START (STrAtegies for RelaTives) intervention for family carers to prevent and manage these symptoms, and found that it is effective and cost‐effective in reducing depression and anxiety in family carers of relatives with dementia in the short term and 6 years later.^3^, ^4^, ^5^, ^6^ It had previously been delivered to family carers around the time of diagnosis, when most people with dementia had very mild, mild or moderate dementia. The intervention is based on a manual and delivered one‐to‐one by supervised, trained facilitators, who are not clinically qualified. It works by addressing the carer's coping strategies and increasing emotion‐focused coping or decreasing the use of dysfunctional coping over time.^7^ The eight sessions comprise behavioural management, communication strategies, identifying and changing unhelpful thoughts, accessing emotional support, increasing pleasant events, relaxation, future planning, and developing a maintenance plan.

Black, South Asians and people from other minority ethnic groups often present to services at a later stage of dementia elated to culturally different understanding of dementia.^8^ Reasons for this include feeling little can be done, previous negative experiences of services, stigma and shame, concerns about the breach of confidentiality, not regarding symptoms of dementia as illness, and a reluctance to reveal private information or share family care with people outside the family.^8^, ^9^, ^10^, ^11^, ^12^, ^13^ This may mean that people with dementia and their families miss out on support while being excluded from services that may feel unavailable or culturally irrelevant.^14^ By the time people contact services, people living with dementia may have higher care needs and families may already be coping with behavioural changes Therefore, ethnicity and culture, as sources of shared norms and values, are important in the dementia care pathway and affect how people with dementia and their families accept or respond to health interventions and cultural adaptation may make them more acceptable Without an intervention being acceptable it cannot be effective but there is reason to think that if people will adhere to a dementia intervention with its core therapeutic elements still incorporated it will continue to be effective.^15^

Although the START intervention and manual have been standardised and made available online with a training programme for trainers,^16^ they have not been specifically adapted or tested specifically with carers of diverse ethnicities and cultures in the UK. In the original trial one‐fifth of participants were from minority groups and they were noted to stop the intervention disproportionately, compared to other groups.^3^ Therefore, we aimed to lay the foundations to widen access to START within the two largest minority ethnic groups of older people in the UK, Black and South Asian groups. In phase one we aimed to identify what aspects of the START intervention need to be culturally adapted for Black and South Asian dementia family carers. In phase two we aimed to test the culturally adapted START intervention for feasibility and acceptability, as well as to consider whether it was as effective as in the original START trial.^15^

## 2 METHODS

We conducted a mixed‐methods study to tailor the START intervention for the UK's South Asian and Black communities' family carers of relatives with dementia and assess its implementation. The study received ethical approval from the London—West London & Gene Therapy Advisory Committee Research Ethics Committee (18/LO/0369). All participants gave written informed consent.

### 2.1 Phase one—Changes to the manual

Family carers were recruited from the London Dementia Network, NHS Memory Services in London which were part of mental health trusts who were identified in trusts and gave consent to be contacted by researchers. We visited Bradford based community organisations to recruit carers. We conducted one‐to‐one qualitative interviews with South Asian (*n* = 15) and Black (*n* = 11) carers. One Patient and Public Involvement (PPI; TB) representative reviewed the topic guides, provided feedback on interview analysis, and made suggestions regarding changes to the manual, such as highlighting that dementia is a disease of the brain rather than something “*mental*” that is “*all in your head*.” We incorporated changes from the interviews in START (carer and therapist version) words and illustrations using the knowledge generated.

We translated START materials in writing and audio‐recording into Urdu (a common South Asian language in the UK) to enable access to those without literacy or those who speak Urdu, not English. A translation agency initially translated a session, but we found that the translation was not appropriately worded, and when asked to retranslate this did not improve. Instead an Urdu‐speaking psychology graduate (SJ) translated it, and NM supervised and edited this. We refined the manuals iteratively through SJ delivering the sessions to NM, which were then audio‐recorded in Urdu. There was no one word for Dementia in Urdu, so we spelt “*Dementia*” phonetically.

### 2.2 Phase two—Intervention delivery and evaluation

We trained three facilitators to deliver START, aiming to deliver to 8–10 South Asian and 8–10 Black carers in English, and three South Asian carers in Urdu. We recruited carers from three NHS trusts memory services in London throughout 2019 who were identified to participate in services after they presented and agreed to be contacted for research. They were then contacted by the research team and gave informed consent to the study. We purposively sampled people with different demographic characteristics, relationship to their relatives with dementia (e.g., spouse or child), and living situations. We collected demographic information from all participants (age, sex, ethnicity, relationship to the person living with dementia, level of education, work and whether they lived with the person with dementia).

The clinically supervised facilitators were two women and one man, all had a psychology degree, one was bilingual and could deliver the intervention in Urdu. We interviewed facilitators after they had stopped delivering START sessions.

We evaluated acceptability through adherence and participants' views of the intervention We defined adherence as attending ≥5 sessions.^3^ We collected views through qualitative interviews using a topic guide (see appendix) after the final START session and 1 year late, also asking if they continued to use the intervention and if so what components. We audio‐recorded and transcribed interviews verbatim and continued until interviews yielded no new substantive information (theoretical sufficiency).

We audio‐recorded a randomly chosen session. Two independent researchers rated the facilitator's delivery fidelity using a session‐specific checklist and gave an overall fidelity score for each session ranging from one to five, with five being high.^3^

We measured the carers' mood at baseline (January–December 2019), immediately post‐intervention (May 2019‐April 2020) and 1‐year post‐intervention (February 2020‐April 2021) using the Hospital Anxiety and Depression Scale (HADS).^17^, ^18^ The HADS‐total score (HADS‐T; possible scores 0–42, higher scores indicating more symptoms) was the primary outcome in the original RCT and aligns with diagnostic criteria for depression. The scale generates the HADS‐depression (HADS‐D) and HADS‐anxiety (HADS‐A); scores from 0 to 21 with ‘caseness’ for scores ≥9. We considered changes in the score post‐study to see if efficacy was in line with the original randomised control trial (RCT).^3^ In the original trial, intervention group carers had a 1.8‐point lower score and a lower risk of case‐level depression (odds ratio 0.24) compared to controls after the intervention.

At baseline, immediately post‐intervention, and 1‐year post‐intervention we measured the carer's quality of life using the Health Status Questionnaire (HSQ)^19^, ^20^ mental health domain. Scores range from zero to 100, with higher scores indicating better QoL.

### 2.3 Analysis

**FIGURE 1:**
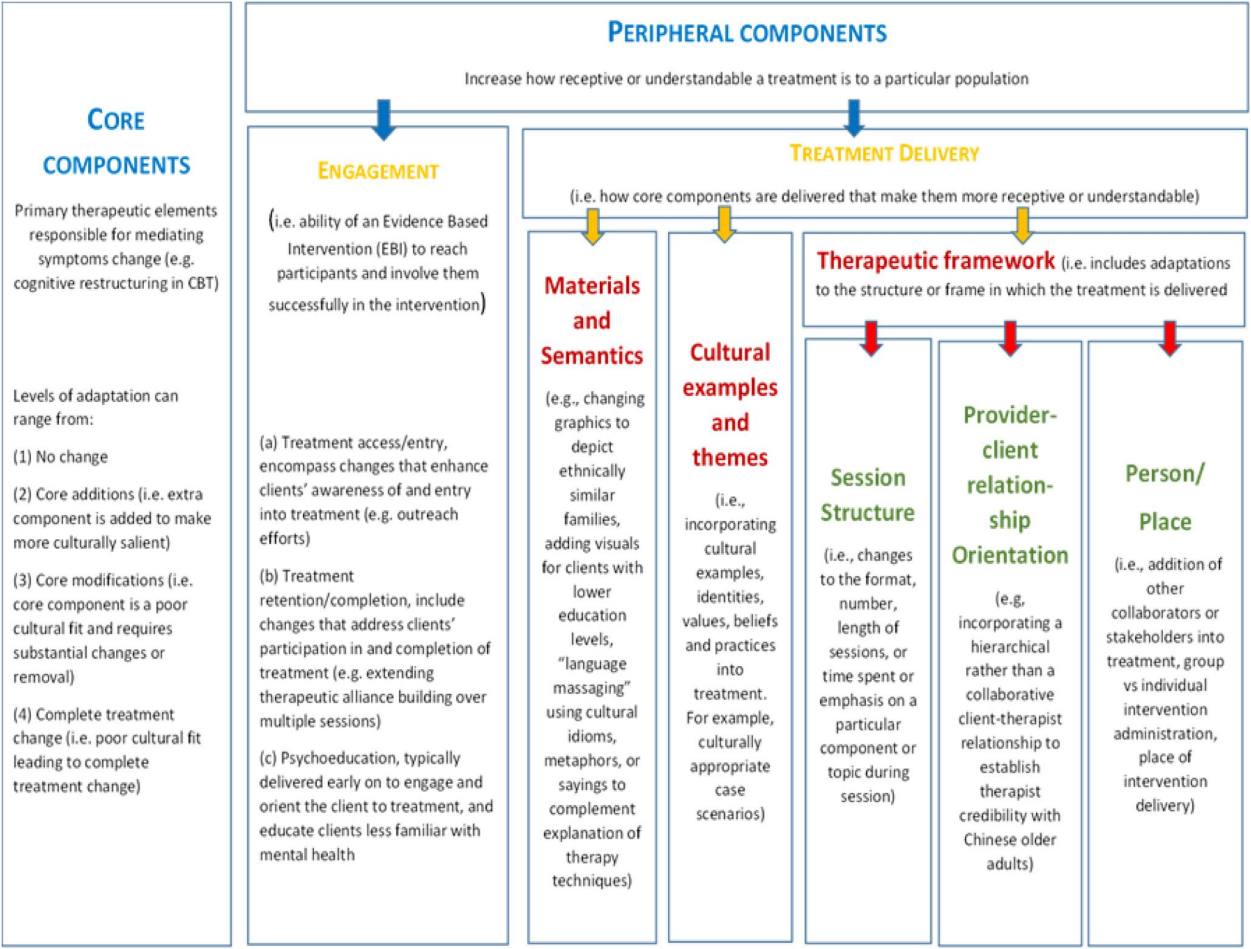
The cultural treatment adaptation framework.

In phase one, two researchers independently double‐coded all phase one qualitative interviews, using thematic analysis. We analysed the interviews about changes using the Cultural Treatment Adaptation Framework (CTAF; Figure 1) to guide the thematic analysis of phase one interviews^21^ covering:

- the core elements of the intervention (e.g., coping strategies, one‐to‐one delivery, relaxation exercises),
- the intervention's engagement, that is, the ability of the intervention to reach participants and involve them successfully in the intervention,
- and the delivery of the intervention, that is, making core components understandable and acceptable.

In phase two, to consider the feasibility and acceptability of the adapted intervention, we described the number of sessions completed and the fidelity. We described the demographics of the carers in the intervention study, the HADS and HSQ mental health score their means, and the differences in mean scores, as well as the number of cases of both anxiety and depression on the HADS, at baseline, immediately post‐intervention, and 1‐year post‐intervention. We planned to consider effectiveness by seeing if, immediately post‐intervention, and 1‐year post‐intervention, the differences in HADS scores between baseline and 1‐year follow‐up were similar to those found in the intervention group in the original START trial (where the HADS total score reduced from 14.8 to 14.3 and the between group difference was 1.8 with odds ratio of 0.25 for depression caseness in the intervention compared to the control group). All descriptive statistics were conducted on SPSS (version 28).

All interviews were independently double coded by two researchers using NVivo (version 12), using thematic analysis. We used the framework developed for the original START trial to guide the thematic analysis of post‐intervention qualitative interviews with family carers.^22^ This frameworks codes the interviews under the main themes of what was helpful or not (from the core components of the intervention), design and form of the intervention, delivery of the intervention, what has been gained or changed, what are they (family carers) are still using, and suggestions for change. For the interviews with intervention facilitators, they were asked about their experiences of when delivering the intervention what went well, what did not go well, and any barriers they had around keeping carers engaged and adhering to the intervention.

## 3 RESULTS

### 3.1 Phase one—Interviews and manual changes

#### 3.1.1 Demographics

In phase one, we interviewed 26 dementia family carers; 15 from South Asian backgrounds and 11 Black carers (see Table 1 for detailed demographics). We recruited people from London or Bradford, of either sex, from a range of countries, with a range of first languages, relationships with the person for whom they cared for and living situations, education levels and employment status. We interviewed all but two South Asian carers in English. We interviewed one carer in Urdu and one in Sylheti with an interpreter.

**TABLE 1:** Carer characteristics (phase one)

Characteristic	South Asian carers (*n* = 15)		Black carers (*n* = 11)	
		*n* (%)		*n* (%)
Age	52 years	Range (25–88)	60 years	Range (43–84)
Sex	Male	6 (40%)	Male	8 (73%)
	Female	9 (60%)	Female	3 (27%)
First language	English	10 (66%)	English	10 (91%)
	Gujarati	1 (7%)	Ibo and English	1 (9%)
	Punjabi	1 (7%)	‐	‐
	Urdu	2 (13%)	‐	‐
	Sylheti	1 (7%)	‐	‐
Ethnicity	Asian/Asian British (Indian)	5 (33%)	African (Nigerian)	2 (19%)
	Asian/Asian British (Pakistani)	3 (20%)	Caribbean (Barbadian,^1^ Grenadian^1^ Jamaican^2^)	4 (36%)
	Asian/Asian British (Bangladeshi)	4 (27%)	British (parents from Antigua,^1^ Barbados,^1^ Trinidad^1^ and Guyana^1^)	4 (36%)
	Any other Asian background	2 (13%)	Mixed (Caribbean and White British)	1 (9%)
	Mixed (White and Asian)	1 (7%)	‐	‐
Relationship to person living with dementia	Spouse	5 (33%)	Spouse	3 (27%)
	Child	8 (53%)	Child	7 (64%)
	Daughter‐in‐law	2 (13%)	Friend	1 (9%)
Co‐resident with the person living with dementia	Yes	9 (60%)	Yes	4 (36%)

Some carers had received START and were familiar with it. We explained the structure to others and asked them to look at one or two sessions in depth.

We did not make any changes to the core components of the intervention but to peripheral elements that render core components more acceptable or understandable, such as materials and semantics, cultural examples and themes, and session structure (see Supplementary File for Table A1 with detailed summary of themes and illustrated them with quotes).

#### 3.1.2 Materials and semantics

Throughout the manuals, we simplified language to increase clarity and allow for lower level of English literacy. We modified graphics to be ethnically diverse, and names in vignette to be ethnically neutral. We referred to carers as “*family member caring for a relative with dementia*” as the term “*carer”* implied for some people being paid to care rather than a relative. When discussing communication strategies, we changed the word “*assertive*” to “*clear.”*

#### 3.1.3 Cultural examples and themes

We added a sentence in the beginning to emphasise cultural competence: “*I may come from a different background to you, and we will have different experiences, but I have experience of working with people from a range of backgrounds*.” We added testimonials around the stressful nature of caring, as several interviewees said that many people in the South Asian community did not acknowledge this. We modified a vignette to illustrate feelings of guilt about “*not being a good daughter*” associated with wanting a break from caring.

#### 3.1.4 Confidentiality and wider family

People from both the Black and South Asian community had concerns about confidentiality as often the dementia diagnosis is not known outside the immediate family, so we added a prompt to highlight that everything said is confidential, as in all health settings. Because more than one family member was likely to be caring, we added a prompt at each session's end: “*What from this session would you want to share with other family members involved in caring for your relative?*” We said that while the same person had to attend every session, some sessions could be attended by other relatives; and provided or directed carers to resources from the voluntary sector to be shared with family members.

#### 3.1.5 Dementia and services

We added more in the section “*overview of memory loss*” highlighting dementia as a “*physical*” illness and not “*just a mental health issue*,” considering the stigma attached to mental health issues. We added a prompt for facilitators when discussing future care options such as admission to a care home, to highlight that “*No one plans to have a member of their family in long‐term care*” but this may sometimes be safest and around short‐term respite options “*as little as an afternoon.”*

### 3.2 Phase two—Delivery of the adapted START intervention

#### 3.2.1 Demographics

**FIGURE 2:**
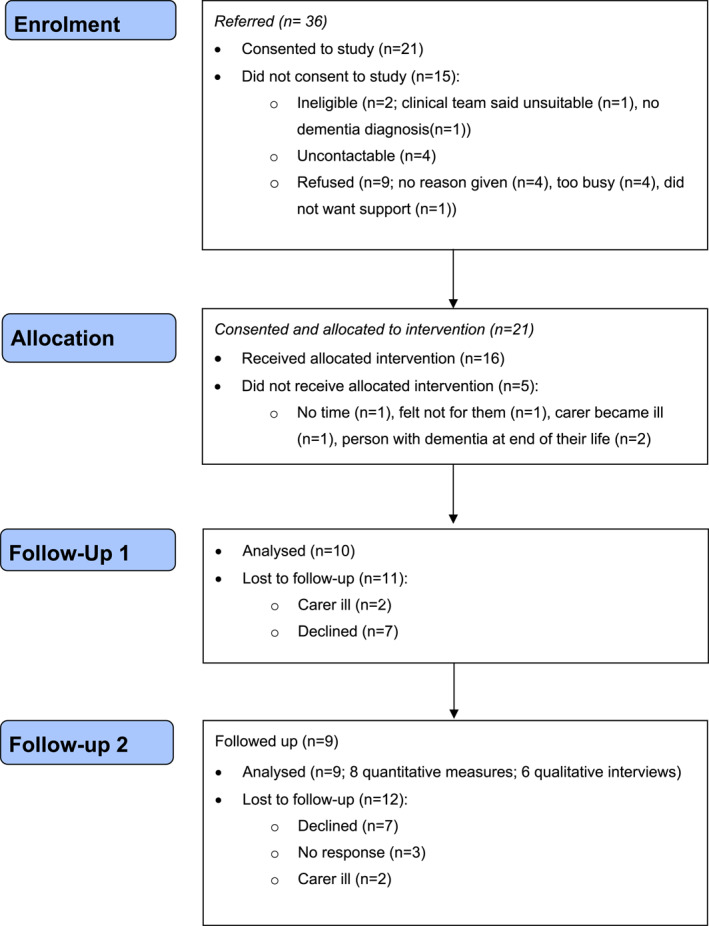
Flow Diagram of study participants.

Thirty‐six carers were referred, 21/30 (70%) of those who were eligible and contactable consented ‐ 13 were South Asian and 8 Black (see Table 2 for carer characteristics, Figure 2 for flow through the study). Five carers withdrew before beginning (4 South Asian, 1 Black).

**TABLE 2:** Carer characteristics (phase two)

Characteristic (*n* = 21)		*n* (%)
Age	55.4 years (SD 15.7)	Range 23–82
Sex	Female	18 (86%)
	Male	3 (14%)
Ethnicity	Indian	7 (33%)
	Pakistani	3 (14%)
	Bangladeshi	2 (10%)
	Burmese	1 (5%)
	African	4 (19%)
	Caribbean	2 (10%)
	Black British	2 (10%)
Marital	Married	15 (71%)
	Divorced	1 (5%)
	Single	4 (19%)
	Widowed	1 (5%)
Education	School level education	10 (48%)
	Further education	11 (52%)
Work situation	Full time	4 (19%)
	Part‐time	8 (38%)
	Not working	6 (29%)
	Retired	3 (14%)
Living with a person with dementia	Yes	18 (86%)
	No	3 (14%)
Relationship with a person living with dementia	Spouse/partner	9 (43%)
	Child	11 (52%)
	Child in‐law/Child's partner	1 (5%)

Adherence was 75% in the 16 carers who began START; 11 completed all sessions (6 South Asian, 5 Black), and one Black carer completed six sessions. The other four carers completed four sessions (South Asian), three sessions (South Asian), two sessions (Black) and one session (South Asian). They stopped because two could not fit sessions around childcare, one wished to attend a group and one was unwell.

We initially delivered all sessions face‐to‐face. The last three participants had sessions seven and eight delivered via telephone while using the START manual for reference, due to the COVID‐19 lockdown in March 2020. We audio‐recorded 12 participants' sessions and their mean fidelity score was 4.4/5 (SD 0.5); four participants were not audio‐recorded as they did not complete the selected sessions.

#### 3.2.2 Follow up of carers who received the adapted START intervention

Immediately post‐intervention, 10/16 carers were followed up with quantitative measures and a qualitative interview. The carers who had not begun START declined follow‐up (4) and the fifth carer was too unwell for an interview. The others were ill (*n* = 1), declined (*n* = 3) or felt unable to complete the follow‐up due to COVID‐19 (*n* = 2).

We interviewed nine carers 1 year after the intervention; eight of whom completed quantitative measures and six completed a qualitative interview, with one carer only completing a qualitative interview. Three of the people not interviewed immediately after the intervention participated in the 1‐year post‐intervention interviews. Twelve carers were not followed up: five who never started the intervention did not want follow‐up, three others declined, three did not respond and one carer was too ill.

#### 3.2.3 Quantitative outcomes

The baseline HADS score amongst all participants (*n* = 21) was 14.4 (SD = 9.8; range 0–33), and for those who started the intervention (*n* = 16) was 12.9 (SD = 8.8; range 0–30). For those who we were able to follow up (*n* = 10) was 12.3 (SD 8.1). Immediately post‐intervention, we interviewed 10 participants and their mean HADS score was 11.3 (SD = 6.1; range 3–19). Nine of them had completed eight sessions and one had completed one. At 1‐year post‐intervention, we interviewed eight participants and their mean HADS score was 12.4 (SD = 6.9; range 5–23).

At baseline, 10/21 (48%) participants who consented were cases of depression on the HADS, seven of whom received the intervention. At the follow‐up immediately post‐intervention, 3/10 (30%) of the people followed up had case levels of depression on the HADS, and 2/8 (25%) at 1‐year post‐intervention. At baseline 8/21 (38%) participants had case‐level anxiety, and immediately post‐intervention and 1‐year post‐intervention this was 3/10 (25%) and 3/8 (37.5%) participants, respectively. The HSQ mental health score changed from a baseline mean of 55.6 (SD 22.9), to immediately post‐intervention 63.3 (SD 20.1), and 59.1 (SD 16.5) 1‐year post‐intervention.

#### 3.2.4 Interviews with intervention facilitators

We interviewed the three intervention facilitators about their experiences in delivering START, including what went well, what did not go well, and any barriers they had around keeping carers engaged and adhering to the intervention.

##### What went well when delivering START

All three described enjoying delivering the intervention, and that they felt it helped them build confidence in delivering interventions and therefore benefitted their career.

Going into a stranger’s home, was a bit scary at first. But I got used to it [and] saw my confidence grow.

(Facilitator 1)

What I enjoyed most probably was the ability to deliver one‐to‐one interventions at an early place in my career. It was supervised, manualised… but. there was a lot of freedom in the session.

(Facilitator 2)

[What] I enjoyed was being able to work with people again… to have face‐to‐face contact with people again… the most rewarding thing was actually seeing that things had changed for people through using START.

(Facilitator 3)

##### What did not go well when delivering START

All three also described challenges with intervention delivery including delivering sessions at home, keeping to timing, gaps between sessions and delivering to carers in Urdu or carers who could not read or write.

But it was more of a difficulty when it came to keeping within the time boundaries and also staying focused on it… often the telephone would ring or there would be someone at the door or a family member will come in.

(Facilitator 2)

I think another big, big challenge, was that one of the carers who can speak Urdu, [was not] able to read or write. And this is an intervention that, the carers have their own [copy] of the manual… to read and to write.

(Facilitator 1)

One facilitator discussed remote delivery at the start of the COVID‐19 pandemic and did not think it was detrimental as they had met participants face‐to‐face previously but it did bring new challenges.

And one of them… used to come to the office, but obviously he couldn’t. They can’t do the sessions in front of the person they care for, so they went and sat in the car, but the exercise was a stretching exercise which just doesn’t work.

(Facilitator 3)

##### Barriers to engagement and adherence of the START intervention

They thought retention of carers may have been difficult, because of time of year for example, religious holidays, carers' personal lives and other responsibilities, and whether the carer feels their relative's dementia was private.

So, I remember when we were recruiting in August. We had Eid, we had Ramadan. And it was quite difficult to keep carers motivated when they had so much… in their personal lives.

(Facilitator 1)

I can only think of one person… that did drop out of the study, and that’s because they were expecting it to be a group, and even though they quite liked the intervention they just wanted to talk to other carers.

(Facilitator 3)

#### 3.2.5 Immediate post‐intervention interviews

Eleven carers completed a qualitative interview immediately after the START intervention.

##### What was helpful or not (from the core components of the intervention)

Different carers valued distinct parts of the intervention most, such as understanding the person with dementia, communicating to their family or the person with dementia, identifying and changing unhelpful thoughts, planning for the future, relaxation techniques, and behaviour strategies. START reminded carers that the behaviours observed in their relative with dementia were a consequence of the illness rather than the person's character. For some carers, the START sessions helped them find better ways to discuss dementia and its progression with their family, a challenging endeavour they had postponed for some time.

That’s what I managed to gain from the sessions. That it is an illness and it’s not her fault.

[carer 1; daughter, South Asian ethnicity].

That’s not a thing you want to talk about… what’s going to happen and stuff. So, it was a bit scary, but I got through it. I thought it was helpful.

[carer 2; daughter, Black ethnicity]

It was comforting to know they had a framework and places where to find help to address future difficulties that might surface. Some carers found that using strategies identified during the session led to fewer difficulties in managing their relative's behaviour.

I know that you can’t really plan for the future because you don’t know exactly what’s going to happen, but you can… have some plans… and that gives me the confidence.

[carer 3; son, Black ethnicity]

And they worked, they actually worked… after about week five, things stabled out.

[carer 4; son, South Asian ethnicity]

Participants described recognising their anger or frustration increasing their relative's difficult behaviour or did not improve it and taking steps to change. They also altered their communication, such as not asking too many questions. Most carers found relaxation strategies useful and shared them with family members and their relatives with dementia. Although, one carer felt uncomfortable engaging in meditation.

I have changed… I used to… come home and… take out all my anger at him… You have to change the words, how you say it. And he listened and we don’t have arguments.

[carer 5; wife, South Asian ethnicity]

Relaxing was the key thing, I really liked START.

[carer 5; wife, South Asian ethnicity]

Because a lot of the meditation stuff I didn’t really want to do.

[carer 6; daughter, Black ethnicity]

##### Design and form of the intervention

Carers commented that the manuals themselves made things less awkward, providing something to look at and work through in the sessions. The examples within the manuals were well‐received and relevant.

You could … relate to the quotes and … discuss a bit more.

[carer 4; son, South Asian ethnicity]

What was really encouraging for me, was that the examples of the scenarios, and the possible solutions, were things that I was already doing… at least I seem to be doing something right.

[carer 3; son, Black ethnicity]

##### Delivery of the intervention

The ethnicity of the facilitator and their cultural competence mattered to some participants as they felt that some challenges were culturally driven and could be better understood by the facilitator who shared that knowledge. Other carers felt that talking to someone outside their cultural group was easier, or the facilitator's ethnicity did not make a difference.

I think just understanding the culture helped and also, she was so friendly… she made it really easy to talk to her… she got it.

[carer 7; daughter, South Asian ethnicity]

And the fact that she’s white British… gave me the permission to say whatever I wanted… people outside of my community are less judgmental.

[carer 3; son, Black ethnicity]

Some participants liked the opportunity to get out of the house, avoiding them talking about their relatives in their own home or neighbours' judgement while others liked the convenience of the intervention being delivered in their home. All participants were positive about the duration and number of sessions. Everyone felt the timing in relation to the diagnosis was suitable and early on after diagnosis seemed to be most appreciated. Some people forgot to do the between sessions task and it was a habit they had to form over time. Other carers found it useful from the beginning. Some carers found it difficult to find time for START for example, because of childcare and cover for their relative's care. Others found that making the commitment was helpful.

It was very useful, because I think without the homework element to it, I probably would have just left it.

[carer 6; daughter, Black ethnicity]

##### What has been gained or changed

Carers felt the intervention changed their understanding, and ultimately the way they communicated and behaved. They also found encouragement to use other services going forward if needed, a renewed motivation for their caring role, and they shared it with other family members.

You have to change the words, how you say it. And he listened and we don’t have arguments, and he comes straight when I say that.

[carer 5; wife, South Asian ethnicity]

I don’t know if things have changed for us, but I’m certainly now more motivated. I’m now doing research. I’m looking into the possible ways of how I can get help, how to make things easier for myself.

[carer 3; son, Black ethnicity]

##### What are they (family carers) are still using

Family carers reported still using a variety of the techniques and components of the intervention. Sometimes this involved going back to the manuals, and other times carers reported using the techniques to work through problems in their minds without needing to go back to the manuals. Carers also reported that there were barriers to continuingly using the techniques, such as feeling too busy.

Not examples, but they’ve got basically a diary of events towards behaviour, either my behaviour or my mum’s behaviour. Did something happen? What was the trigger? How did you respond? I don’t write that down, but I do do it in my head, if that makes sense.

[carer 8; daughter, Black ethnicity]

##### Suggestions for change

Overall, there were a few changes suggested. They were around more specific directions, more complex examples, and more cultural examples in the manuals.

To me, they were very simplistic. And I know that you can’t put real‐life scenarios in there, but maybe they needed to be a little bit more taxing. I don’t know if that’s the right word. But I found them over simple, if that makes sense.

[carer 8; daughter, Black ethnicity]

#### 3.2.6 One‐year post‐intervention interviews

Six carers completed a qualitative interview around 1‐year after they received START.

##### What was helpful or not (from the core components of the intervention)

Overall, the carers interviewed discussed how the START intervention had made them feel less alone in their caring role and had had a positive impact on them and the person they were caring for 1 year later. Carers also felt that the intervention came at the right for them.

I found out I wasn’t alone, and I learnt these techniques… From then I was comfortable dealing with… If you don’t get any help, that’s where people like me get frustrated and depressed.

[carer 9; daughter, Black ethnicity]

But, luckily, because I was doing START, I feel like it was the exact right time for me to do it. I was able to figure out, okay so now she’s upset. She’s angry. She’s stressed. Going through the booklets and use what have to do to help her calm down.

[carer 7; daughter, South Asian ethnicity]

There were particular strategies carers discussed finding helpful, including the relaxation techniques and the behavioural management strategies.

The relaxation thing I absolutely love… The last time I used it for myself was a couple of months ago. But, it is something that I was using quite a lot before. I still have it on my phone just in case.

[carer 7; daughter, South Asian ethnicity]

Because before, when he’s angry I’m also angry but I started not to be at the same time as him. I started to calm myself down so that was… Because if I don’t, we both argue a lot, especially I’m supposed to know better at times, but since I read the hand‐out, so I started focus and go through it again.

[carer 9; daughter, Black ethnicity]

##### Design and form of the intervention

The main comments around the design and form of the intervention were being able to go back to the manuals and look at the examples, suggestions, and questions to work through and record notes.

Whatever it was, for whatever reason, being able to record it. Write it down when it happened.

[carer 7; daughter, South Asian ethnicity]

##### Delivery of the intervention

At the 1‐year post‐intervention, carers described that they preferred the one‐to‐one delivery of the intervention at the time they received it, but a year on they may have preferred a group setting, due to the progression of their relative's dementia and also because they felt ready to share their experiences.

Because at that particular time I think that’s what I needed. I think maybe later, in terms of a group, I have now find a group situation more advantageous now, because it’s like now I’m ready to share the experience.

[carer 8; daughter, Black ethnicity]

##### What has been gained or changed

Some carers said they shared components of the manuals with other relatives or friends who were caring for people living with dementia. One carer also discussed how they now had a social worker and a paid carer helping to look after their relative as a direct result of the intervention.

My wife has used some of the stuff as well. We’ve gone through it, and my daughters as well.

[carer 4; son, South Asian ethnicity]

I’m at the age now where a lot of people… are facing the same kind of issues with their parents… well they don’t have access to those services. They, sometimes they feel at a bit of a loss and I’m able to… give them ideas that I learnt with … It has been helpful.

[carer 3; son, Black ethnicity]

##### What are they (family carers) are still using

All discussed continuing to use at least one component of the START intervention. These included relaxation exercises, communication techniques, developing behavioural strategies, and increasing pleasant events.

That’s one of the big things that’s really helped. Just ask her. So, I do ask her. I go away, and I come back… Then she would give me an answer.

[carer 7; daughter, South Asian ethnicity]

I do yoga three, three times a week., I go for walks. I listen to… music. So, these… help me calm down.

[carer 2; daughter, Black ethnicity]

The carers also spoke about some components they had stopped using within the year post intervention, often the record forms and written plans. This was either because they had forgotten or used components in ways that suited them, for example, on their phones or working through the steps mentally.

The main one is the continued use of the behaviour record. Not because I don’t want to, because it was … very helpful. But just because I’ve forgotten. I keep the list. It all stays in a file.

[carer 7; daughter, South Asian ethnicity]

I don’t always write it down, but I do work it out in my head.

[carer 8; daughter, Black ethnicity]

##### Suggestions for change

The main suggestions for change at the 1‐year post‐intervention point was with the examples within the manuals being too simple, which was also a suggested immediately post‐intervention, and that sometimes the examples weren't relevant because they were for people with more severe dementia.

I know that they’re quite simple and they obviously don’t cover every scenario. But yes some of them I thought were maybe too simple, does that make sense?

[carer 8; daughter, Black ethnicity]

I found some of the stuff in the workbook, the scenarios that they give weren’t relevant for me and my mum because I’m not a full‐time carer. And she didn’t need a full‐time carer.

[carer 8; daughter, Black ethnicity]

## 4 DISCUSSION

This is to our knowledge the first study to tailor and test an intervention for family carers of people affected by dementia in UK‐dwelling Black and South Asian communities.^15^ We had envisaged that we would produce different tailored manuals for each of the groups, however after the changes suggested we did not feel this to be necessary. We found that the changes suggested, such as making names culturally neutral and pictures ethnically diverse, emphasising confidentiality, that caring was hard, and that facilitators were culturally competent, could benefit people from different backgrounds. We are now using this culturally adapted version of START for everyone. This is because as the interviewees did not suggest changing the core components, which are those we regard as therapeutic, we do not expect these changes to impact the effectiveness of the intervention although it might improve the acceptability in all groups.^15^

Fidelity to the intervention was high. Of those who began sessions, 75% were adherent suggesting the intervention was acceptable. This is higher adherence than in the original START trial, where 61% of minority ethnic carers attended ≥5 sessions.^3^ However, this study had a higher rate of participants not starting the intervention (23%) compared to the original trial where this was 5%.^3^

Several people who asked to postpone for Ramadan never began or found that childcare responsibilities during school holidays prevented them from starting or continuing attendance. The difference in mental health and quality of life post‐intervention were in line with the original study, suggesting effectiveness. All carers reported continued use of at least one component from the START intervention immediately post‐intervention and 1 year later. Qualitative feedback was consistent with that of the original START clinical trial ‐ that START improved their ability to cope, they continued to use some strategies and could refer to the manual to remind themselves of helpful practices.^22^

We translated the START intervention into Urdu, and it was difficult to develop a good culturally appropriate translated version. This increased access to the START intervention for people from the South Asian community as Urdu is a commonly spoken language in the UK, but it may change cost‐effectiveness to translate into lesser‐used languages. To further widen access to the START intervention we have worked with appropriate teams to translate it into Hindi, Spanish and Japanese, and for use in India in English, Tamil and Kannada.

The themes in the study did not appear to be unique to minority ethnic carers but some were more common in these groups. They wanted facilitators with cultural competence, the intervention delivered with consideration of their other caring and religious duties and had concerns about privacy and confidentiality. Familism was more common, with carers for example, more frequently than our experience has been in the majority white population prioritising their duty to look after family members, who are children, for their parent in school holidays, over an intervention to help them cope. Again, this occurs in all groups. The ethnicity of the facilitator was important to some carers, but some people wanted carers of the same and some of different ethnicity. We have not asked this question of white UK carers.

### 4.1 Strengths and limitations

We were able to interview people with a range of demographic characteristics and reach theoretical sufficiency although there will always be some views that are not captured. Our qualitative analysis used a culturally appropriate framework and two independent raters for reliability. We were, however, only able to follow up with 10 people recruited after the intervention, and 1 year later this dropped to nine. As seven out of 10 of those who had case level depression completed the intervention we know that it was acceptable both for some people who were depressed and some who were not. This was partly related to the beginning of COVID‐19 when people stopped services, were unused to communicating online, including filling in questionnaires, or the person with dementia and carers were ill. We do not know if those not seen were systematically different.

For the qualitative interviews in both phases, the researchers who collected and analysed the data were from a variety of cultural backgrounds, including sometimes similar backgrounds to the participants who were interviewed. They considered the effect their ethnicity and gender had and as laid out some participants were explicit that they preferred someone of the same characteristics as themselves and others liked them to be different. Overall the researchers were t a mix of insider and outsider researchers, and there are pros and cons to both positions,^23^ including in terms of minimising bias.

We used the HADS and HSQ to measure both anxiety and depression and health status, and both of these questionnaire have been used in studies with dementia carers previously.^24^, ^25^ We used the English versions of both measures which may be a limitation, and though each, particularly the HADS, has been translated and validated in other languages and used within different cultures, the evidence on their cross‐cultural validity is scarce.^26^

We had no control group in this trial, unlike the original RCT where the control group's mental health deteriorated, thus accentuating the between‐group difference. Nevertheless, it is appropriate to use an acceptability and feasibility trial rather than a full controlled trial is not necessary to test an intervention that has not modified its therapeutic components.^15^

## 5 CONCLUSIONS

The culturally adapted START intervention for family carers from Black and South Asian British backgrounds has the potential to help underserved UK populations. It was feasible and acceptable, with similar effectiveness to the original trial. Clinicians should consider the calendar for religious holidays, childcare, and other caring responsibilities when they offer START. There are many differences within as well as between ethnicities and the START changes address issues relevant to the majority population, so the changes benefit all ethnicities. This is now the available online version irrespective of ethnicity.

## CONFLICTS OF INTEREST

Gill Livingston, Andrew Sommerlad, Naaheed Mukadam and Penny Rapaport are supported by the University College London Hospitals' National Institute for Health Research (NIHR) Biomedical Research Centre (UCLH NIHR BRC). Gill Livingston is supported by the National Institute for Health Research ARC North Thames and as an NIHR senior investigator. Moïse Roche is funded by Alzheimer's Society UK (404 AS‐PhD‐17b‐007). The views expressed in this publication are those of the author(s) and not necessarily those of the funders or National Institute for Health Research or the Department of Health and Social Care.

## Supporting information

Table S1

## Data Availability

The datasets generated by this study are available upon request from the corresponding author.
