# Supplementary material for: Tailoring STrAtegies for RelaTives for Black and South Asian dementia family carers in the United Kingdom: A mixed methods study
Source: Int J Geriatr Psychiatry. 2023 Jan 15;38(1):e5868. doi: 10.1002/gps.5868 (PMC10107806; doi:10.1002/gps.5868)
Supplement: Supplementary file 1 [file GPS-38-0-s001.odt]

Table A1: Summary of changes to manual following phase one interviews 

|  | Change suggested | Quote | Action taken |
| --- | --- | --- | --- |
| Peripheral components Treatment Delivery Materials and Semantics | Audio versions of START | …would really help people who can’t read and aren’t able to write. You would reach a lot more people. | Already in protocol |
| Peripheral components Treatment Delivery Materials and Semantics | START materials and intervention to be available in other languages | If you don’t speak English, seeing a picture of an Asian person is one thing, ... it feels very, sort of, inclusive, but then reading in your own language … is a lot more inclusive. | Already in protocol |
| Peripheral components Treatment Delivery Materials and Semantics | Family members of relatives with dementia may not self-identify as “carers” understanding the term as paid carers, using carer for administrative forms. Use “family member caring for a relative with dementia.” | I did identify [as a carer, I use it].  For paperwork but ... I identify as mum’s daughter… the carer thing... I just don’t like it, ... it’s so weird. I think growing up just hearing carer is someone that comes in and they’re paid. | Change Terminology: instead of the term “carer” use “family members caring for a relative with dementia” |
| Peripheral components Treatment Delivery Materials and Semantics | Would not use “South Asian” but would use “Asian” + specific country |  | Instead of South Asian use “Asian family carers and e.g., Bangladesh |
| Peripheral components Treatment Delivery Materials and Semantics | No word for dementia in South Asian languages. The term “dementia” can be used if spelled phonetically in Urdu so that it sounds like “dementia” when read aloud. | I think there needs to be more awareness of Dementia within our communities, I mean there isn’t even a word for Dementia in the Urdu, Punjabi, and Bengali languages. | Spell dementia phonetically in Urdu |
| Peripheral components Treatment Delivery Materials and Semantics | Use of the word “assertive” potentially problematic | Assertive communication wouldn’t work in our communities.  ... we can’t speak to our husbands that way.  ... It would be seen as disrespect. | Instead of the word “assertive,” use the term, firm or clear |
| Peripheral components Treatment Delivery Materials and Semantics | Adapt for older generations or carers with lower education levels | The manual ... lends itself to more formal educated background. ...People will probably need …help to navigate. | Ensure language is clear and not complex |
| Peripheral components Treatment Delivery Materials and Semantics | Changing graphics to be more culturally appropriate | I mean there's quite a few images that are … Two white people shaking hands and nothing wrong in that, but most of the imagery assumes they're white | Change/Include images to be more inclusive |
| Peripheral components Treatment Delivery Cultural examples and themes | Role of carer not recognised; caring for older people a cultural norm and expected; Stressful nature of caring not well understood within the community | The whole stress on the body thing. It’s a difficult one because where caring for the elders isn’t seen as you are doing anything out of the ordinary, the person you care for, and family members don’t understand the toll. Yes, another bit is relief really. I find that it’s okay to feel stress, but a bit of the South Asian culture is ...my parents took care of me when I was young, so it is my duty and part of my privilege to care for them. Yes, I don’t think people really realise the impact caring has on carers. | Incorporate into sessions by modifying examples to illustrate this, including excerpts from interviews with SA carers in Phase one who have received START that acknowledges this |
| Peripheral components Treatment Delivery Cultural examples and themes | Session 6 – Planning for the future; respite care, long-term care may not be considered acceptable options within the community | .. some of the things that are offered wouldn’t be relevant. Like, the respite services. That was something I discussed with XXXXX, but the respite services that are available, it’s not something that mum would actually partake in. | In session 6: ask what they already know about care options Highlight that care options need not be long-term; respite care can be for e.g., an afternoon Add in sentence: “No one plans to have a member of their family placed in long-term care” |
| Peripheral components Treatment Delivery Cultural examples and themes | Incorporating something around dealing with conflict of feelings of guilt associated with not wanting to care for a relative, which goes against the sense of duty and expectations of, for example, a “good” daughter | And it was a case of, okay, well, you’ve got nothing to feel guilty about, it’s like, yes, but it’s my obligation. And she’s like, no it’s not your obligation. I was like, yes, this is the thing that someone not from the South Asian community wouldn’t understand | Session 4 vignette modified to explicitly illustrate someone who does not want to care all the time and the resulting sense of guilt, “not doing their duty”. session 5: Example about difficulties of asking for help, when children should see it as a “privilege” to care. |
| Peripheral components Treatment Delivery Therapeutic framework Session structure | Education about Dementia needed (i.e., biological changes) | I think it’s tough because I don't think they really see it as an illness ... they just see not like as a problem but more like a mental ... I think unfortunately there is not a proper understanding of what dementia and Alzheimer’s and all that is within our community in general I mean everything in the community is a taboo | Add in a prompt at the end of each session (orange box) “What from this session would you want to share with other family members involved in caring for your relative?” Provide or direct to information leaflets from the Alzheimer’s Society about illness  (Translated as needed) |
| Peripheral components Treatment Delivery Therapeutic framework Provider client relationship orientation | People outside the immediate family often not aware that a family member has dementia, so would need to take this into consideration when organising START sessions (e.g., option to have START the home or local community) | A big thing for my mum ... other family members knowing. No-one outside of our immediate family really knows. Within the Asian community... everyone has a doctor in the extended family. So, a lot of people would just tap into their community. | Emphasise respecting confidentiality in session 1 Also, in “Planning for the future,” discussion point around how information is shared/respecting confidentiality |
| Peripheral components Treatment Delivery Therapeutic relationship Provider client relationship orientation | No consensus on whether it should be someone with the same cultural background delivering but importance of facilitator understanding the culture | I don't really mind, but I think it's important to have someone who understands me, and who understands everything that I'm saying. | Added a sentence to session 1 (facilitator has experience of working with people from a range of backgrounds) |
| Peripheral components Treatment Delivery Therapeutic framework Person/Place | Multiple family members involved in caring for the person with dementia. Carers want to be able to share information and learning with them - handout for information about dementia and how it can affect the relative and the carer (e.g., stress), also how to share strategies developed with family members | I think they’d be positive about it because I’d share that information that I’ve learnt ... with my brothers, my mum, everybody.  ... And they’d realise, if she’s doing that, it must be good, that this is the plan, and this is what might happen if I'm not here, but you guys just need to be aware of I have three other siblings, so I was filtering down that information... | Add in a prompt at the end of each session (orange box) “What from this session would you want to share with other family members involved in caring for your relative?” Provide information leaflets (preferably translated into South Asian Language) |
| Peripheral components Treatment Delivery Therapeutic framework Person/Place | Often large and close-knit families potentially all involved in caring for the person living with dementia. | You know, in the south Asian communities, ... they're very - what do you call – close-knit families. And they may be interested in it… some of them may be interested | Add at the beginning of manual, that it is important that one person attends ALL sessions but some sessions can be attended by more than one family member |
| Peripheral components Treatment Delivery Therapeutic relationship Person/Place | Because people outside immediate family sometimes unaware of diagnosis, ideally want option for delivery of START from someone outside local community. | I don’t know how I would feel if it was somebody from within my own community, within my own area, that came and spoke to us. If it was somebody maybe outside, then I guess that would be more accepting. | Emphasise respecting confidentiality in session 1. Also, in Session 6 “Planning for the future”, discuss respecting confidentiality |
| Peripheral components Treatment Delivery Therapeutic relationship Person/Place | Flexibility in place to deliver START (e.g., at home or outside the home). | think some [sessions] should be at home where the person with dementia is included … or at least be privy to what’s being discussed, and some ..away so that the carer does have that time where they just get themselves out of the situation. | Flexibility there by design |
| Peripheral components Treatment Delivery Therapeutic relationship Person/Place | Delivering to more than one family member BUT not at the same time | I want my sons and my daughters to understand and be involved.... because it means that we'll all understand it. It might be too heavy if it was more than one or two people in a session. | Invite other family members for session 6 and prompt “how would you want to share this information with members of your family” |
